# Rbfox1 Is Expressed in the Mouse Brain in the Form of Multiple Transcript Variants and Contains Functional E Boxes in Its Alternative Promoters

**DOI:** 10.3389/fnmol.2020.00066

**Published:** 2020-05-05

**Authors:** Sonia Casanovas, Laura Schlichtholz, Sophia Mühlbauer, Sri Dewi, Martin Schüle, Dennis Strand, Susanne Strand, Lea Zografidou, Jennifer Winter

**Affiliations:** ^1^Institute of Human Genetics, University Medical Center Mainz, Mainz, Germany; ^2^Focus Program of Translational Neurosciences, University Medical Center Mainz, Mainz, Germany; ^3^First Department of Internal Medicine, University Medical Center Mainz, Mainz, Germany; ^4^German Resilience Centre, University Medical Center Mainz, Mainz, Germany

**Keywords:** *Rbfox1*, alternative splicing, neurodevelopmental disorders, autism, alternative promoters

## Abstract

The RNA-binding protein RBFOX1 is an important regulator of neuron development and neuronal excitability. *Rbfox1* is a dosage-sensitive gene and in both mice and humans, decreased expression of *Rbfox1* has been linked to neurodevelopmental disorders. Alternative promoters drive expression of *Rbfox1* transcript isoforms that encode an identical protein. The tissue- and developmental stage-specific expression of these isoforms, as well as the underlying regulatory mechanisms, are, however, unclear. Here, we set out to capture all of the Rbfox1 transcript isoforms and identify transcriptional mechanisms that regulate brain-specific *Rbfox1* expression. Isoform sequencing identified multiple alternative *Rbfox1* transcript variants in the mouse cerebral cortex, including transcripts with novel first exons, alternatively spliced exons and 3′-truncations. Quantitative RT-PCR determined the expression of the alternative first exons in the developing cerebral cortex and different subregions of the juvenile brain. Alternative first exons were found to be highly stage- and subregion specific in their expression patterns suggesting that they fulfill specific functions during cortex development and in different brain regions. Using reporter assays we found that the promoter regions of the two first exons E1B and E1C/E1C.1 contain several functional E-boxes. Together, we provide an extensive picture of *Rbfox1* isoform expression. We further identified important regulatory mechanisms that drive neuron-specific *Rbfox1* expression. Thus, our study forms the basis for further research into the mechanisms that ensure physiological *Rbfox1* expression in the brain. It also helps to understand why, in patients with neurodevelopmental disorders deletion of individual *RBFOX1* transcript isoforms could affect brain function.

## Introduction

The RNA Binding Fox-1 Homolog 1 (*Rbfox1*) gene is one of the longest genes in the genome with its remarkable size being caused by an extended 5′-noncoding region containing several alternative 5′UTR exons, transcription of which is controlled by alternative promoter usage (Kuroyanagi, 2009; Damianov and Black, 2010; Conboy, 2017). An important yet unanswered question is why so many alternative *Rbfox1* transcripts exist that encode an identical protein. These transcripts could theoretically encode RBFOX1 proteins associated with specific functions. Alternatively, tissue-specific transcription factors might bind the alternative *Rbfox1* promoters and fail-safe *Rbfox1* expression levels in a tissue-specific manner.

RBFOX1 is a member of the RBFOX family of RNA binding proteins that also includes RBFOX2 and RBFOX3. *Rbfox1* expression is restricted to neurons, heart, and muscle (Shibata et al., 2000; Jin et al., 2003; Underwood et al., 2005). In the cell, RBFOX1 shuttles between the nucleus and cytoplasm, an event which is regulated by alternative splicing of the exon A53. Splicing of exon A53 into the *Rbfox1* mRNA causes cytoplasmic localization of RBFOX1 by introducing a frame-shift and an alternative C-terminus lacking the nuclear localization signal (NLS; Underwood et al., 2005; Lee et al., 2009). In the nucleus, RBFOX proteins regulate alternative pre-mRNA splicing by binding the consensus sequence (U)GCAUG in introns flanking alternative exons. Cytoplasmic RBFOX1 influences gene expression, by binding the 3′-untranslated regions (3′UTRs) of its target genes (Dredge and Jensen, 2011; Lee et al., 2016; Rajman et al., 2017).

RBFOX1 is linked to the etiology of neurodevelopmental disorders. Thus, studies in knockout mice have shown that in the brain, RBFOX1 controls splicing and expression of transcripts involved in neuronal transmission and excitability (Gehman et al., 2011; Vuong et al., 2018; Wamsley et al., 2018). Subsequent studies identified multiple additional RBFOX1 splicing targets in differentiating human fetal neural progenitor cells and in mouse brain (Fogel et al., 2012). These targets included many genes related to autism spectrum disorders (ASD). For example, the vSNARE protein VAMP1 was identified as a major RBFOX1 target in inhibitory neurons of the hippocampus (Vuong et al., 2018). Re-expression of *Vamp1* rescued the electrophysiological abnormalities seen in the *Rbfox1* knockout mice. Furthermore, *RBFOX1* was identified as a hub gene in ASD transcriptional networks. The downregulation of *RBFOX1* observed in autistic brains was reflected by RBFOX1-dependent splicing changes (Parikshak et al., 2013).

Copy number variations (CNVs) in the *RBFOX1* gene are associated with various neurodevelopmental disorders such as epilepsy, intellectual disability (ID) and ASD (Bhalla et al., 2004; Martin et al., 2007; Lal et al., 2013; Zhao, 2013). A characteristic feature of these CNVs is their location in the *RBFOX1* gene, which is typically restricted to the long 5′-noncoding part of the gene. Each of the CNVs described so far in patients with neurodevelopmental disorders affect some, but not all, of the alternative *RBFOX1* transcripts. Since it is unknown at which developmental stages and in which tissues every single alternative transcript is expressed and how each transcript contributes to the overall *RBFOX1* expression levels it remains unclear to what extent these CNVs change overall *RBFOX1* expression levels in the brain.

Deeper insight into the expression patterns of the alternative *Rbfox1* transcripts and their transcriptional regulation through alternative promoter usage is key for the understanding of how the RBFOX1 function is controlled in the healthy and diseased brain. In this study, by performing Isoform sequencing (Iso-Seq) and RT-qPCR we have characterized in detail which alternative *Rbfox1* transcripts are expressed in the embryonic and postnatal cerebral cortex and the juvenile mouse brain. Besides *Rbfox1* transcript variants already known before, we have identified several novel transcripts some of which contain novel coding exons. These exons are located even further upstream of the known Rbfox1 5′ exons and encode a unique N terminus with a predicted classic NLS. We could confirm the protein expression of these isoforms and found that they localize to the cell nucleus even if they lacked the C-terminal NLS. To investigate the transcriptional regulation of *Rbfox1* we have searched for transcription factor binding sites in the alternative *Rbfox1* promoters. We have found that two of the alternative *Rbfox1* promoters contain functional E-boxes that promote *Rbfox1* expression in primary neurons.

## Materials and Methods

### Ethics Approval

Sacrificing mice to obtain brain tissue was performed according to European (EU directive 86/609/EEC), national (TierSchG), and institutional guidelines.

### Nomenclature of Gene and Protein Names

Throughout the manuscript, we followed the rules of the Human Genome Organization Nomenclature Committee^1^ and the Mouse Genome Informatics Nomenclature Committee^2^.

### Mice

NMRI (12 weeks old for neuron culture, 6 weeks old for isolation of brain subregions) mice from Janvier labs (Saint Berthevin, France) were sacrificed by cervical dislocation upon arrival. For the collection of brain subregions from juvenile mice, brains were removed and incubated in RNAlater^®^ (Sigma, Munich, Germany) for 2 days at 4°C. After dehydration, brain regions were dissected and further processed. Three male mice were used for expression analyses. For neuron culture, cortical neurons were isolated from E14.5 embryos from pregnant female NMRI mice.

### Plasmids, *in vitro* Mutagenesis

*Rbfox1* 1B (positions −2,147 to +1,039), 1C (positions −989 to +470) and 1D (positions −2,302 to +56) promoter fragments were PCR amplified from mouse DNA and cloned into the multiple cloning site of pGL4.10 firefly luciferase reporter vector using KpnI and HindIII (Rbfox1 1B) or XhoI and BglII (Rbfox1 1C and 1D). Site-directed mutagenesis was performed using the QuikChange Multi Site-Directed Mutagenesis Kit (Agilent Technologies, Waldbronn, Germany) and following the manufacturer’s recommendations. Primer sequences are given in Supplementary Table S1.

Rbfox1 isoforms starting from E1A with exon A53 skipped or included were PCR amplified from total RNA of P0 mouse cerebral cortex with primers containing XmaI and EcoRI overhangs and cloned into the multiple cloning sites of the pCAGGs-IRES-GFP vector. The accuracy of cloned constructs was verified by Sanger sequencing. Primer sequences are given in Supplementary Table S1.

### Cell Culture, Reporter Assays and Immunofluorescence Experiments

P19 cells were cultured in DMEM high glucose supplemented with 10% FBS, 1% NEAA and 100 μg/ml Penicillin/Streptomycin. N2A cells were cultured in DMEM high glucose supplemented with 10% FBS and 100 μg/ml Penicillin/Streptomycin. Primary cortical neurons, prepared from E14.5 mouse embryos, were cultured in neurobasal medium containing 2% B27 supplement (Gibco Life Technologies, Carlsbad, CA, USA) and 500 μM Glutamax (Gibco Life Technologies, Carlsbad, CA, USA). See Supplementary Methods for a detailed description of luciferase assays and immunofluorescence experiments.

### RT-qPCR

Total RNA was extracted from mouse brain tissue using TRIzol (Thermo Fisher Scientific, Rockford, IL, USA) and from primary neurons and P19 cells using the High Pure RNA Isolation kit (Roche, Basel, Switzerland). Reverse transcription was performed with the RevertAid First Strand cDNA Synthesis kit (Thermo Fisher Scientific, Rockford, IL, USA). Quantitative PCR was performed on an ABI StepOnePlusTM Real-Time PCR System using Sybr Green or Rox Mix (Applied Biosystems, Carlsbad, CA, USA). The Universal Probe Library Technology (UPL; Roche, Basel, Switzerland) was used to directly compare the expression of different transcripts. Primer efficiencies were calculated with the software LinRegPCR (Ruijter, 2004). Relative expression was calculated using the equation published by Pfaffl (2001) which adjusts for the primer efficiency. Primer sequences are given in Supplementary Table S1.

### Iso-Seq Sequencing and Analysis

For isoform sequencing total RNA from the cerebral cortex of a mouse at stage P0 with a RIN value >9 was sent to the Vienna Biocenter Core Facilities (VBCF) of the Vienna Biocenter. The single-molecule real-time (SMRT) library preparation and sequencing was performed at the VBCF NGS Unit^3^. See Supplementary Methods for a detailed description.

### Data Availability

The binary Alignment/Map files (BAM) from the combined sequencing data were deposited in the NCBI Sequence Read Archive (SRA) under the BioProject accession number PRJNA587931.

### *In situ* Hybridization

Two riboprobes, one corresponding to exon 1B and the other one to a part of the coding sequence of *Rbfox1* were generated and hybridized on E15.5 mouse brain sections (ZYAGEN, San Diego, CA, USA). *In situ* hybridizations were analyzed with an EVOS XL Core Cell Imaging System (Thermo Fisher Scientific, Rockford, IL, USA). See Supplementary Methods for a detailed protocol.

### Design and Statistical Analyses

All statistical analyses were done with GraphPad Prism 5 or Microsoft Office Excel. Data are shown as mean + standard error of the mean. Statistical analyses were done using student’s *t*-test. *p* values < 0.05 were considered statistically significant.

## Results

### Identification of Alternative Rbfox1 Transcripts by Isoform Sequencing

**Figure 1:**
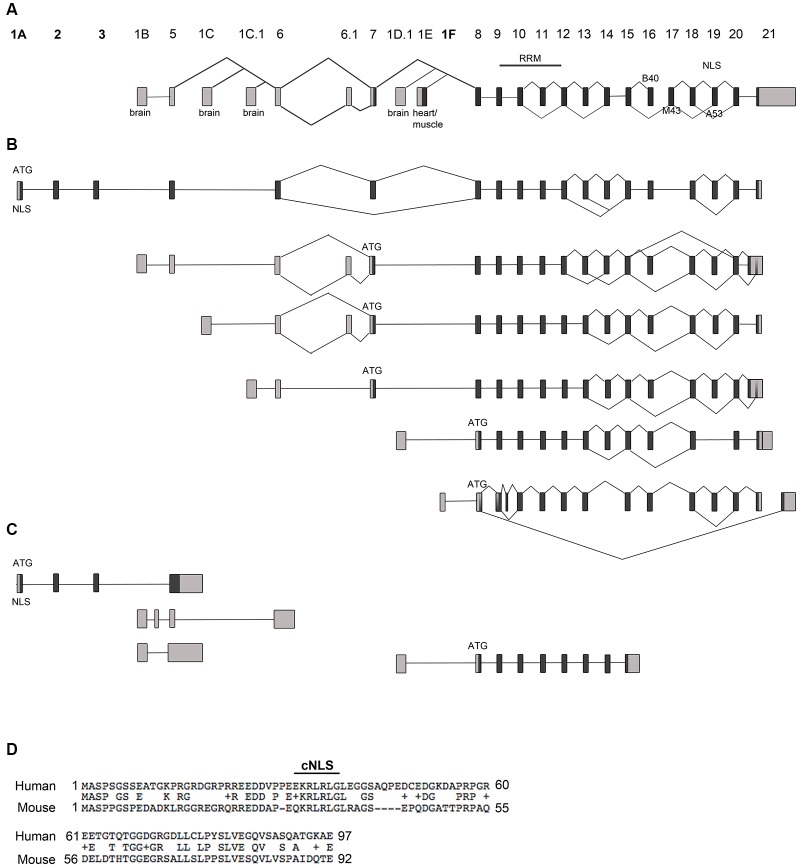
*Rbfox1* transcript identification in the cerebral cortex. **(A)** Scheme of the *Rbfox1* gene with the known exon annotation (Damianov and Black, 2010). **(B,C)** A summary of the alternative *Rbfox1* transcripts expressed in the perinatal cerebral cortex. Total RNA was isolated from P0 mouse cortices and used for Iso-Seq. **(B)** Full-length* Rbfox1* transcripts. **(C)** Three prime truncated *Rbfox1* transcripts. Boxes represent exons. Dark gray boxes indicate coding exons and light gray boxes non-coding exons. Novel exons are named 1A, 2, 3, and 1F. Alternative exons B40, M43, and A53 are shown. RRM, RNA recognition motif; NLS, nuclear localization signal. **(D)** Conservation of the novel exons 1A, 2 and 3 between human and mouse. cNLS, predicted classic nuclear localization signal.

*Rbfox1* is one of the longest genes in the genome with an extensive 5′-region. It is transcribed from several alternative promoters. The complexity of *Rbfox1* transcripts further increases by alternative splicing and alternative polyadenylation events. It is, however, unclear how many different alternative *Rbfox1* transcript isoforms exist and whether there is any preference in alternative exon combinations. To identify all existing full-length *Rbfox1* transcript isoforms in the mouse cerebral cortex, we performed Iso-Seq. Iso-Seq is a relatively recently developed sequencing method that creates long reads and therefore provides sequences of full-length transcripts. Iso-Seq of total RNA from mouse postnatal day (P) 0 cerebral cortex revealed a very complex picture of *Rbfox1* isoforms with multiple transcripts containing alternative first, internal or last exons or alternative polyadenylation sites (PAS; Figures 1A–C, Supplementary Figure S1). Interestingly, in addition to the already known brain-specific alternative first exons E1B, E1C, E1C.1 and E1D (Figure 1A) we identified several novel first exons that are, as the already known alternative 5′-terminal exons, likely to be transcribed from their promoters (Figure 1B, Supplementary Figure S1). All of these additional first exons are spliced to the known *Rbfox1* internal exons and are therefore expected to encode RBFOX1 protein variants. One of these first exons is located 578 kb upstream of E1B and was therefore named E1A. It is spliced to two additional novel exons upstream of E1B, which we named exons 2 and 3 followed by the already known internal *Rbfox1* exons. While it was originally thought that most of the alternative *Rbfox1* first exons are noncoding, the novel exons E1A, 2 and 3 are coding exons and, when integrated into the *Rbfox1* open reading frame, add 92 amino acids to the RBFOX1 N-terminus. The splice sites of these novel exons are conserved in mammals. Their overall conservation at the protein level is, however, low (59.3% identity) between mouse and human. The first exon E1A encodes a predicted cNLS which is conserved in humans (Figure 1D). RBFOX1 isoforms containing E1A are therefore supposed to localize to the nucleus and participate in the alternative splicing process.

Several other novel first exons (E1F, E1F.1, E1F.2) were identified downstream of the heart and muscle-specific first exon E1E (Figures 1A,B; Supplementary Figure S1). As for transcripts starting in E1E, their translational start codon is in exon 8.

The *Rbfox1* gene contains a long 3′-untranslated region (3′UTR) of 3.7 kb with several alternative PAS. Almost all *Rbfox1* transcript isoforms identified here were polyadenylated at an alternative PAS located 150 bp downstream of the translation termination codon, which suggests that Rbfox1 transcripts in the P0 cerebral cortex typically contain a short 3′UTR.

In addition to alternative 5′ and 3′ splicing, alternative splicing of internal exons were observed in the Iso-Seq data. Inclusion of the alternative *Rbfox1* exon A53 introduces a frame-shift in the C-terminus and causes a lack of the C-terminal NLS with the consensus sequence FAPY. These protein isoforms are therefore expected to be mostly cytoplasmic. Transcription of a given alternative first exon was generally not associated with specific splicing events. Alternative splicing of exon A53, for example, was a frequent event in the P0 cerebral cortex and occurred in transcript isoforms containing the alternative first exons E1A, E1B, E1C, E1C.1 and E1F (Figure 1B). Likewise, alternative splicing of the brain-specific exon B40 and *Rbfox1* exons 13 and 14 was frequently observed. Similar to the inclusion of exon A53 the skipping of exon B40 introduces a frame-shift at the C-terminus.

In addition to the full-length transcripts, we identified several 3′-truncated transcripts (Figure 1C). Most of them do not contain an open reading frame of substantial length.

### Rbfox1 Isoforms Carrying the Alternative First Exon 1A Are Translated Into Protein and Localize to the Nucleus

Nuclear localization of Rbfox1 is regulated by alternative splicing of exon A53. Inclusion of this exon causes a frame-shift and a lack of the C-terminal NLS and has been shown to guide Rbfox1 to the cytoplasm (Lee et al., 2009). Rbfox1 isoforms starting from the novel exon E1A contain a predicted cNLS which theoretically might ensure nuclear localization of Rbfox1 isoforms lacking the C-terminal NLS.

**Figure 2:**
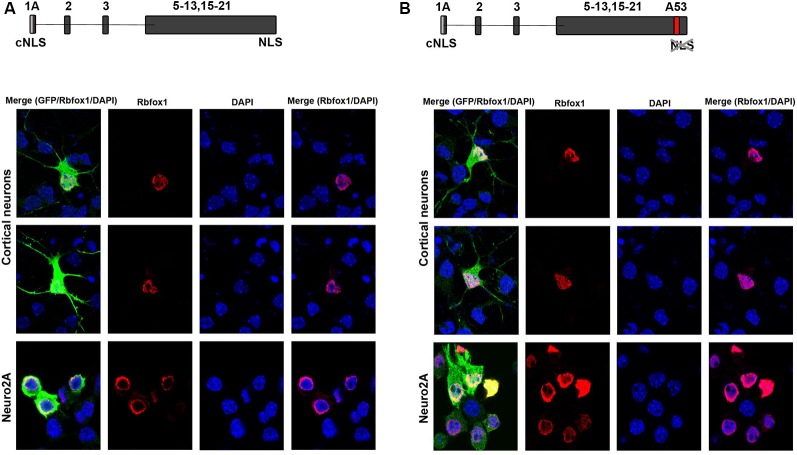
Expression of novel Rbfox1 isoforms starting from exon 1A in N2A cells and primary neurons. **(A,B)** Immunofluorescence analysis of the subcellular localization of the two Rbfox1 isoforms starting from exon 1A. N2A cells or primary cortical neurons were transfected with constructs encoding the untagged Rbfox1 isoforms followed by an IRES GFP. Twenty-four hours later cell were fixed and immunofluorescence stainings were performed with antibodies specific for Rbfox1 and GFP. **(A)** Construct encoding Rbfox1 isoform starting from exon 1A and lacking exon A53. **(B)** Construct encoding Rbfox1 isoform starting from exon 1A and containing exon A53.

To test for protein expression and subcellular localization of Rbfox1 isoforms starting from E1A we generated two different constructs, the first encoding untagged Rbfox1 isoform 1A without exon A53 and the second untagged Rbfox1 isoform 1A with exon A53 included. Both constructs also contained an IRES GFP downstream of the Rbfox1 coding sequence to detect transfected cells. We chose untagged Rbfox1 constructs because a tag at the N-terminus might bias expression of the Rbfox1 open reading frame while a tag at the C-terminus might hinder the NLS which is encoded by the last four amino acids. After transfecting these constructs into Neuro2A (N2A) cells and primary cortical neurons we performed immunofluorescent stainings with Rbfox1 and GFP specific antibodies. We could not detect the expression of endogenous Rbfox1 in N2A cells suggesting that expression levels of Rbfox1 are either absent or too low for antibody detection in this cell line. Although we could detect endogenous Rbfox1 expression in primary cortical neurons using the Rbfox1 specific antibody the signal was rather weak and could be clearly distinguished from the Rbfox1 signal of the overexpressing neurons (Figures 2A,B). The immunofluorescence stainings revealed nuclear localization of Rbfox1 regardless of whether exon A53 was included in the respective constructs or not (Figures 2A,B).

### Expression of Rbfox1 Isoforms Carrying Alternative First Exons in the Pre- and Post-natal Cerebral Cortex and in the Juvenile Brain

**Figure 3:**
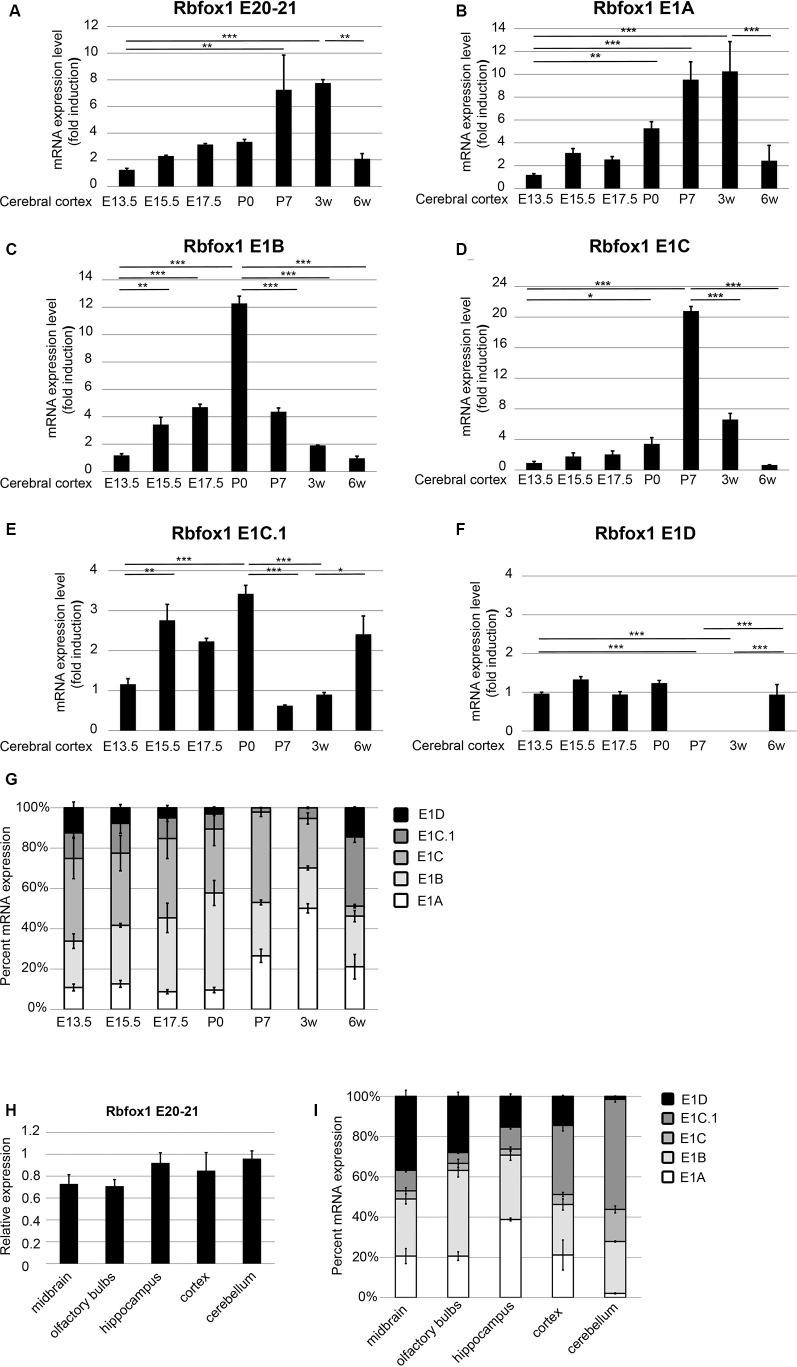
Expression of the *Rbfox1* alternative first exons during embryonic and postnatal development of the cerebral cortex and in different brain subregions. **(A–F,H)** RT-qPCR with primers corresponding to *Rbfox1* exons 20/21 or the alternative first exons. Total RNA was isolated from the cerebral cortex at different embryonic/postnatal stages **(A–G)** and different brain subregions of 6-weeks old mice **(H,I)**, and cDNA synthesis and qPCR were performed; mRNA expression was normalized to Gapdh. **(G,I)** Direct comparison of the expression levels of the alternative *Rbfox1* first exons. The sum of the expression levels of all alternative first exons was set to 100%. Data represent the average of three (juvenile brain regions) to four (embryonic/postnatal stages) biological replicates. The standard error of the mean is indicated by black bars. Statistical analyses were done using one-way-ANOVA with Bonferroni’s multiple comparison test. *P < 0.05; **P < 0.01; ***P < 0.001.

To further study the expression of the *Rbfox1* alternative first exon transcripts during cortical development we performed RT-qPCR experiments with RNA from the cerebral cortex of mice at embryonic days (E) 13.5, 15.5, 17.5, P0, P7, and at 3 and 6 weeks of age. To cover the expression of the majority of *Rbfox1* transcripts within a single RT-qPCR reaction we generated primers located in the *Rbfox1* exons 20 and 21. This primer combination amplifies all *Rbfox1* transcripts except the 3′-truncated transcripts and weakly expressed *Rbfox1* transcripts that are terminated at an alternative PAS upstream of exon 21 (Supplementary Figures S1, S2). Using these primers, we observed a 7.8-fold increase in *Rbfox1* expression from E13.5 until 3 weeks of age (Figure 3A) whereafter Rbfox1 expression decreased strongly (3.7-fold from 3 weeks to 6 weeks of age). To analyze the expression levels of the alternative first exons and their contribution to overall *Rbfox1* expression levels we then generated primers corresponding to sequences in these exons. Expression of most of these alternative first exons also increased from E13.5 until perinatal or postnatal stages and declined thereafter. We observed an increase in expression until P0 for E1B until P7 for E1C and until 3 weeks of age for E1A (Figures 3B–D). After reaching these peaks expression of these first exons declined and at 6 weeks of age reached levels similar to or slightly above the levels observed at E13.5. E1C.1 expression increased from E13.5 to P0, decreased from P0 to P7 and increased again thereafter (Figure 3E). By contrast, *Rbfox1* 1D expression was stable from E13.5 to P0, undetectable at P7 and 3 weeks of age and increased again at 6 weeks of age (Figure 3F).

Since, we used probe-based qPCR assays we were able to compare the transcript levels of the different *Rbfox1* isoforms directly after correcting for differences in primer efficiencies (see “Materials and Methods” section). A direct comparison of the expression levels of the different *Rbfox1* first exons showed that E1B and E1C/E1C.1 together accounted for approximately 80% of the total amount of all measured *Rbfox1* first exon variants in the E13.5 to P0 cerebral cortex (Figure 3G). *In situ* hybridization experiments confirmed the expression of *Rbfox1* E1B in the cortical plate of E15.5 embryos (Supplementary Figure S3). From P0 to P3 weeks of age the percentage of E1A in the total amount of expressed first exons increased from 9.5% to 50% and declined to 22% at 6 weeks of age.

To test how *Rbfox1* alternative first exon isoforms contribute to total *Rbfox1* expression levels in juvenile brain regions we carried out RT-qPCR with RNA from the midbrain, olfactory bulbs, hippocampus, cerebral cortex, and cerebellum of 6-weeks old mice. We found that while the total *Rbfox1* levels (detected with primer combination in exons 20/21) are relatively constant throughout different brain regions (Figure 3H) some of the alternative first exons are expressed in a brain region-specific manner (Figure 3I; Supplementary Figure S4). In the hippocampus, E1A was the predominant first exon variant and accounted for 40% of the total amount of all measured *Rbfox1* first exon variants. In the cerebellum, the expression of E1A was very low. E1B expression levels were relatively constant in all brain regions examined with a 2-fold higher expression in olfactory bulbs and hippocampus compared to other brain regions. *Rbfox1* E1C/E1C.1 was expressed most strongly in the cerebellum (71% of all measured *Rbfox1* first exon variants) whereas *Rbfox1* E1D was highly expressed in the midbrain (37% of all measured *Rbfox1* first exon variants).

### Several E-Boxes Regulate Neuronal Expression of Rbfox1 Isoforms 1B and 1C/1C.1

**Figure 4:**
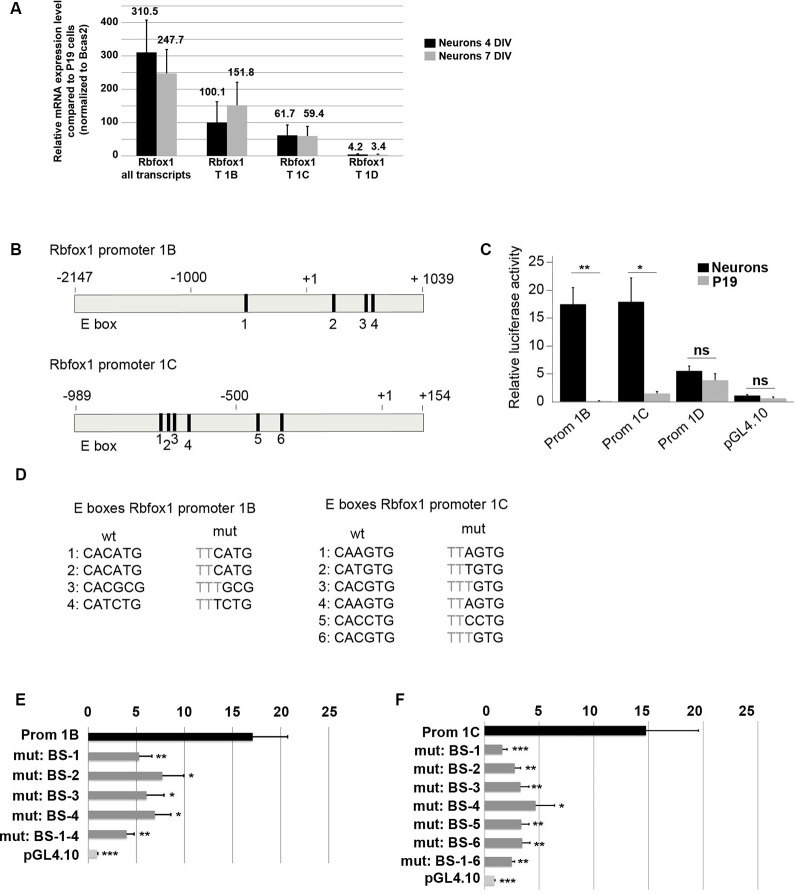
E-boxes promote expression of *Rbfox1* 1B and 1C/1C.1 in neurons. **(A)** Expression of *Rbfox1* alternative first exons in primary cortical neurons. Total RNA was isolated from primary cortical neurons that had been in culture for 4 or 7 days, and cDNA synthesis and qPCR experiments with primers corresponding to *Rbfox1* exons 20/21, 1B, 1C or 1D were performed (*n* = 4). **(B)** The scheme of the *Rbfox1* 1B and 1C/1C.1 upstream regulatory regions that were cloned into the pGL4.10 vector. Black boxes represent evolutionary conserved E-boxes. Numbers indicate the nucleotide position relative to the transcriptional start sites. **(C)** Luciferase assays were performed in primary cortical neurons and undifferentiated P19 cells to determine the promoter activities of the *Rbfox1* 1B, 1C/1C.1 and 1D upstream regulatory regions (*n* = 8). Statistical analyses were done using student’s *t*-test. ns, not significant; *P < 0.05; **P < 0.01. **(D)** Sequences of the wildtype and mutated E-boxes as depicted in **(B)**. **(E,F)** Luciferase assays were performed to determine the activity of the *Rbfox1* 1B and 1C/1C.1 upstream regulatory regions with or without mutations in all E-boxes or every single E-box (*n* = 6). The standard error of the mean is indicated by black bars. Statistical analyses were done using one-way-ANOVA with Bonferroni’s multiple comparison test. *P < 0.05; **P < 0.01; ***P < 0.001.

The first exon variants E1B and E1C/E1C.1 are the most strongly expressed variants in the developing cerebral cortex (Figures 3C–E,G). This suggests that the promoter regions driving expression of E1B and E1C/E1C.1 are highly active in this region. To further confirm this we cultured primary cortical neurons from E14.5 mouse embryos and measured expression of E1B, E1C/E1C.1 and for comparison E1D. As expected, these neurons expressed *Rbfox1* exons E1B and E1C/E1C.1 strongly and exon E1D only weakly (Figure 4A). To assess whether the regions upstream of exons E1B, E1C/E1C.1 and E1D contain neuron-specific promoters we cloned these regions into the pGL4.10 luciferase vector and carried out luciferase experiments in cortical neurons (Figures 4B,C). All three regions produced significantly more luciferase activity than the empty vector (Figure 4C). Also, the regions upstream of E1B and E1C/E1C.1 produced significantly more luciferase activity in primary neurons than in undifferentiated P19 cells which do not express endogenous *Rbfox1*. They also produced three times more luciferase activity than the region upstream of E1D suggesting that these regions are more active in cortical neurons derived from embryonic tissue. We therefore concentrated on these regions in subsequent experiments.

A visual inspection of the cloned regions upstream of E1B and E1C revealed several evolutionarily conserved E-boxes (Figures 4B,D); four in the region upstream of E1B and six in the region upstream of E1C. We could not detect conserved E-boxes in the upstream regions of any of the other *Rbfox1* first exon variants. E-boxes are well described binding sites for bHLH transcription factors. To test whether the E-boxes upstream of E1B and E1C were functional, we mutated each of these binding sites individually. Also, we generated constructs carrying mutations in all of these binding sites. Mutating the E boxes upstream of E1B and E1C caused a significant drop in luciferase activity for each of the single mutations (Figures 4E,F). This suggests that the E boxes upstream of E1B and E1C are all functional and might act together to drive transcription of E1B and E1C.

## Discussion

Haploinsufficiency of the *Rbfox1* gene causes neuronal hyperexcitability and seizures in *Rbfox1* knockout mice (Gehman et al., 2011). In humans, heterozygous CNVs in the 5’-noncoding part of the *RBFOX1* gene that typically only affect some but not all *RBFOX1* transcript isoforms are associated with a range of neurodevelopmental disorders (Bhalla et al., 2004; Martin et al., 2007; Zhao, 2013). While this indicates dosage sensitivity for *Rbfox1*, regulatory mechanisms that balance *Rbfox1* expression levels are unclear. One reason for this might be the size and complexity of the *Rbfox1* gene which suggests complex modes of regulation. In this study, we identified all *Rbfox1* transcript variants detectable by Iso-Seq in the mouse cerebral cortex at P0, and characterized the alternative *Rbfox1* promoters.

Here, we show that transcripts starting in the novel first exon E1A encode a unique N-terminus containing a predicted cNLS, which is conserved in humans. RBFOX1 contains a C-terminal NLS of the sequence FAPY, which is typical for nuclear RNA binding proteins (Sun et al., 2012). Skipping of exon B40 or inclusion of exon A53, however, leads to a frame-shift, an alternative C-terminus lacking the FAPY NLS and cytoplasmic localization of the encoded RBFOX1 protein isoforms (Lee et al., 2009). RBFOX1 protein isoforms containing the N-terminal NLS brought in by exon E1A however localized to the nucleus even if exon A53 was included in the respective *Rbfox1* transcripts. Strong expression of E1A as seen in the cerebral cortex at 3 weeks of age and in the hippocampus at 6 weeks of age might be a guarantee for correct splicing patterns through a high concentration of RBFOX1 in the nucleus. We have recently found a similar phenomenon in the *Rbfox2* gene, which contains an N-terminal cNLS in its isoform 1A (Wenzel et al., 2016). *Rbfox2* protein isoforms with this cNLS and lacking the FAPY NLS in the C-terminus are indeed nuclear.

Skipping of exon 11 produces a dominant-negative RBFOX1 isoform, which has been found in muscle and heart but not in the brain (Damianov and Black, 2010). In agreement, we did not detect *Rbfox1* transcripts lacking exon 11 in P0 cerebral cortex. This suggests that skipping of exon 11 is a heart and muscle-specific event and does not play a role in the brain, at least not in the regions and stages tested here. Rather, we found frequent skipping of exon 14 in the P0 cerebral cortex, a splicing event, which to our knowledge has not been described before. Skipping of exon 13 was detected as well although in fewer transcripts. Skipping of exons 13 and 14 leads to in-frame deletions of 18 and 27 amino acids, respectively, in the RBFOX1 protein. Both exons encode parts of the C-terminus of RBFOX1, which interacts with multiple binding partners including several RNA binding proteins and is crucial for exon activation and repression (Sun et al., 2012). Skipping of exons 13 and 14 could therefore theoretically change the binding of RBFOX1 to its interactors or influence the ability of RBFOX1 to activate and/or repress target exons.

In mammals, and eukaryotes in general, splicing is coupled to transcription (Kornblihtt, 2015). Not only the speed of transcription can pre-determine alternative splicing events but also the expression from different promoters (Tasic et al., 2002). In our study, we could not observe that expression from alternative *Rbfox1* promoters was linked to skipping or inclusion of alternative internal exons. For example, we could detect both skipping and inclusion of exon A53 in *Rbfox1* transcripts expressed from almost all of the alternative promoters. This suggests that alternative promoters in the *Rbfox1* gene do not regulate alternative splicing choices. However, Iso-Seq is not a quantitative measure of transcript abundance and it might be that the ratio between skipped and included alternative exons varies in transcripts generated from the different promoters. Despite we cannot exclude this possibility our results rather suggest that the many different promoters in the *Rbfox1* gene are needed to contribute to total *Rbfox1* levels in different developmental stages and brain subregions. This is supported by the observation that expression levels of total *Rbfox1* were relatively constant between different brain subregions whereas those of the alternative transcripts were stage- and region specific. It must, however, be mentioned that the analysis of brain regions with high cellular heterogeneity is a limitation of this study.

The usage of alternative promoters may theoretically be linked to specific RBFOX1 functions as has been observed for other proteins (Bae et al., 2014; Zhu et al., 2014; Philips et al., 2015; Perera and Kim, 2016). A similar mechanism has been described for the *BDNF* gene, which contains nine alternative promoters driving the expression of identical BDNF proteins (Maynard et al., 2016). Knockout studies in which BDNF production from each one of the four major promoters was disrupted provided evidence that *BDNF* transcripts produced from different promoters mediate different aspects of behavior. Whether this is the case for the alternative *Rbfox1* transcripts as well is not clear. In humans, CNVs that are located in different parts of the 5’-region of the *RBFOX1* gene have been found in patients with ID, ASD, ADHD, epilepsy, and schizophrenia (Bhalla et al., 2004; Martin et al., 2007; Elia et al., 2010; Lal et al., 2013; Zhao, 2013). These CNVs span 5′-UTR exons of unique *RBFOX1* transcript isoforms. They are therefore predicted to interfere with the expression of some but not all *RBFOX1* transcripts. If the location of these CNVs was correlated with specific phenotypes in the patients this might be an indication of transcript specific functions. This is, however, not the case. Rather, CNVs spanning overlapping regions have been observed in patients with variable phenotypes and patients with similar phenotypes carry CNVs spanning distinct regions in the *RBFOX1* gene (personal observation). Approximately 50% of the genes that are linked to neurological disorders are transcribed from alternative promoters, and specific transcript variants are involved in these diseases. It may, therefore, be important to study the expression and functions of these gene isoforms instead of concentrating on the whole gene’s function (Pal et al., 2011). For *Rbfox1*, future studies should aim to generate transcript specific knockouts in mice or human cell models.

## Data Availability Statement

The raw data supporting the conclusions of this article will be made available by the authors, without undue reservation, to any qualified researcher.

## Ethics Statement

Ethical review and approval was not required for the animal study because the study does not include any animal experiments requiring approval. To carry out this study mice were killed for organ removal. In Germany, this procedure is notifiable but does not require approval by an ethics committee.

## Author Contributions

SC and LS designed and performed experiments and analyzed the data. SM, LZ, and MS performed experiments and analyzed the data. SD, DS, and SS analyzed the data. JW planned experiments, analyzed the data and wrote the manuscript. All authors read and approved the final manuscript.

## Conflict of Interest

The authors declare that the research was conducted in the absence of any commercial or financial relationships that could be construed as a potential conflict of interest.

## Footnotes

^1^https://www.genenames.org/data/gene-symbol-report/#!/hgnc_id/HGNC:18222

^2^http://www.informatics.jax.org/marker/MGI:1926224

^3^www.vbcf.ac.at

## Supplementary Material

FIGURE S1 Rbfox1 transcript identification in the cerebral cortex. All spliced Rbfox1 transcript variants that were detected by Iso-Seq in the P0 cerebral cortex are shown. At the top of each page is a schematic of the known Rbfox1 exons (18). RRM, RNA recognition motif; NLS, nuclear localization signal; pA, polyadenylation site; ATG, translational start codon.

FIGURE S2 Expression of the alternative 3′end of the Rbfox1 gene during cortex development. Total RNA was isolated from the cerebral cortex of different embryonic and perinatal stages and subjected to RT-qPCR with primers corresponding to Rbfox1 exons 20/21 or the alternative 3′ end (*n* = 3); mRNA expression was normalized to Gapdh.

FIGURE S3 Rbfox1 first exon 1B is expressed in the cortical plate of the mouse embryo. **(A)** *In situ* hybridization of Rbfox1 (genepaint.org) showing Rbfox1 expression in the cortical plate, and several other brain regions. **(B,C)** *In situ* hybridization carried out with probes corresponding to total Rbfox1 **(B)** or Rbfox1 1B **(C)**. A high expression level of Rbfox1 1B was detected in the cortical plate.

FIGURE S4 Expression of the Rbfox1 alternative first exons in different subregions of the juvenile brain. RT-qPCR with primers corresponding to Rbfox1 exons 20/21 or the alternative first exons. Total RNA was isolated from different brain subregions of 6-weeks old mice, and cDNA synthesis and qPCR were performed; mRNA expression was normalized to Gapdh.

TABLE S1 Primer sequences.
