# Supplementary figures and images for: Rbfox1 Is Expressed in the Mouse Brain in the Form of Multiple Transcript Variants and Contains Functional E Boxes in Its Alternative Promoters
Source: Front Mol Neurosci. 2020 May 5;13:66. doi: 10.3389/fnmol.2020.00066 (PMC7214753; doi:10.3389/fnmol.2020.00066)

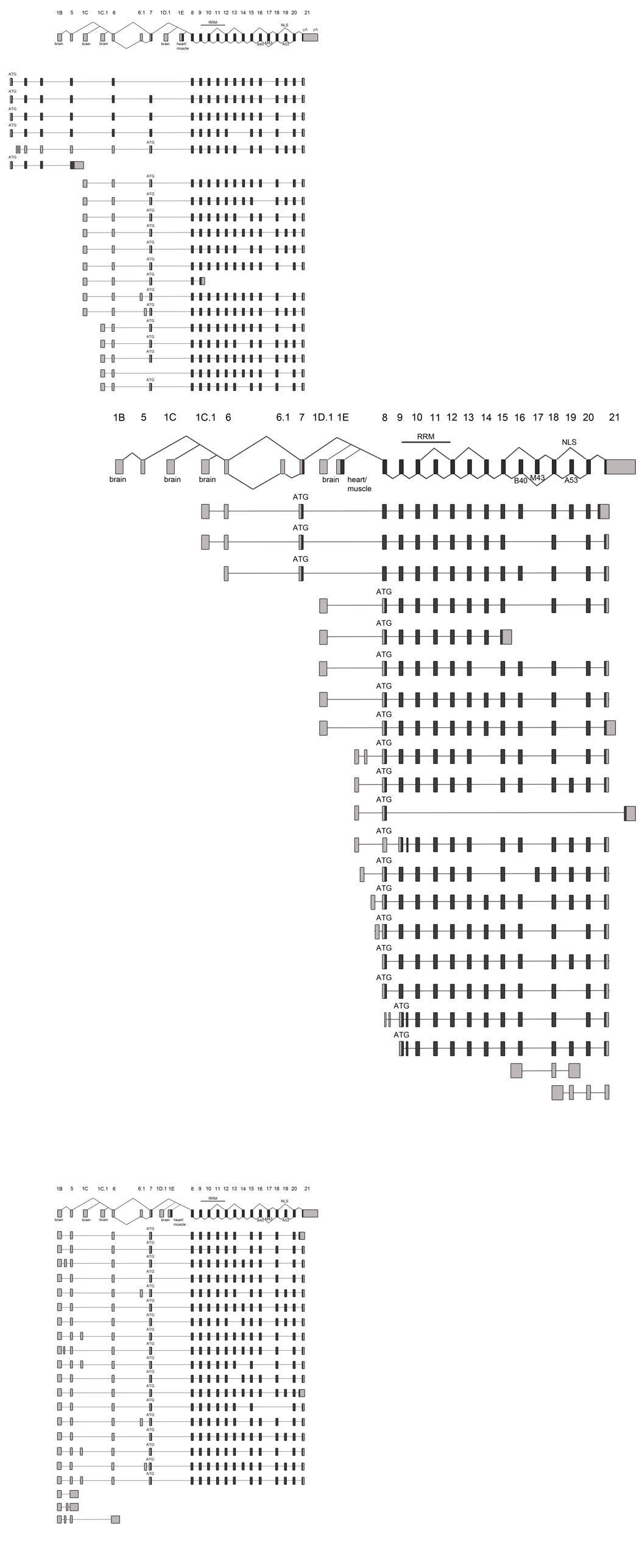

Supplement: FIGURE S1 — Rbfox1 transcript identification in the cerebral cortex. All spliced Rbfox1 transcript variants that were detected by Iso-Seq in the P0 cerebral cortex are shown. At the top of each page is a schematic of the known Rbfox1 exons (18). RRM, RNA recognition motif; NLS, nuclear localization signal; pA, polyadenylation site; ATG, translational start codon. [file Image_1.jpg]

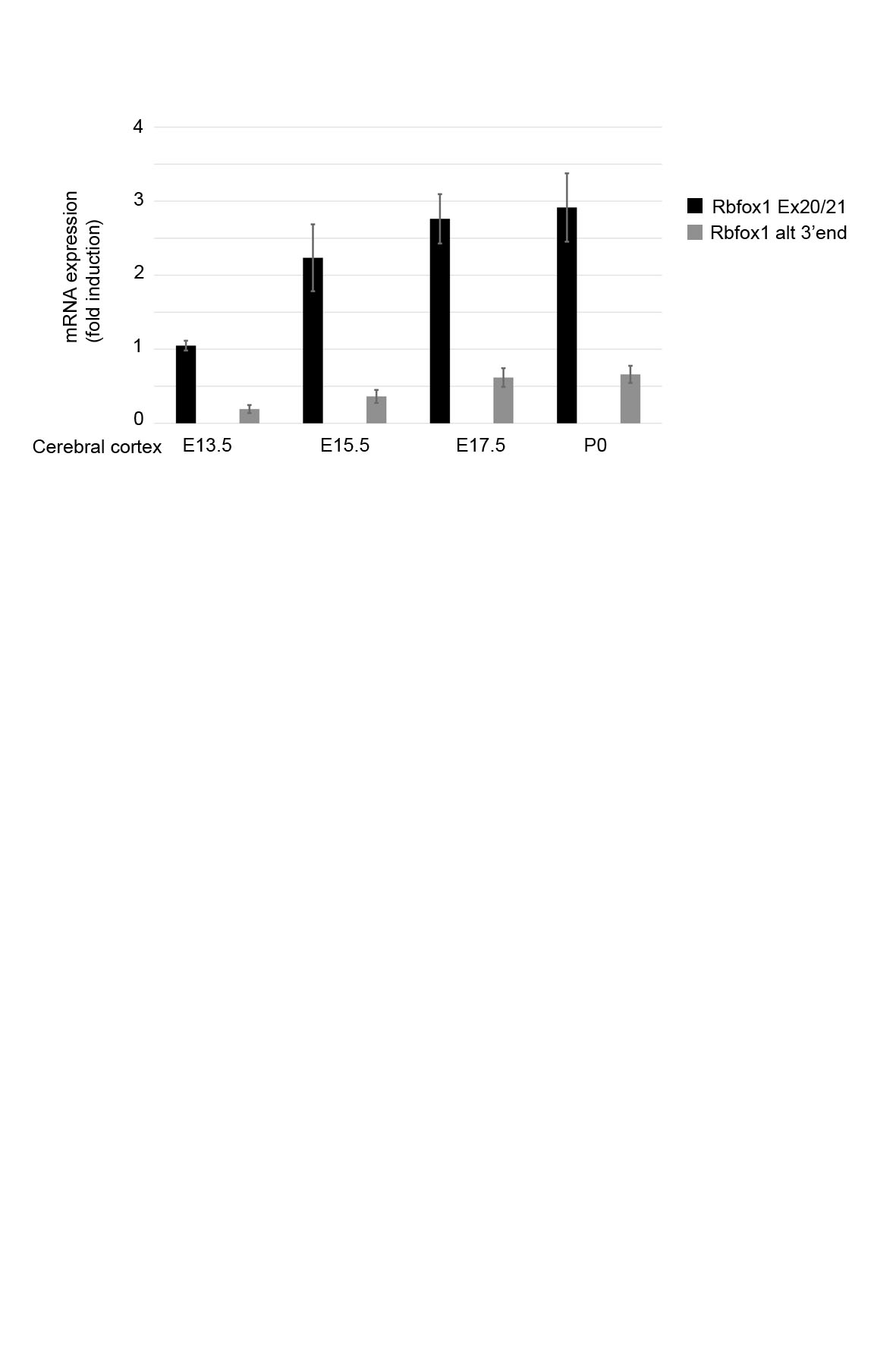

Supplement: FIGURE S2 — Expression of the alternative 3′end of the Rbfox1 gene during cortex development. Total RNA was isolated from the cerebral cortex of different embryonic and perinatal stages and subjected to RT-qPCR with primers corresponding to Rbfox1 exons 20/21 or the alternative 3′ end (n = 3); mRNA expression was normalized to Gapdh. [file Image_2.jpg]

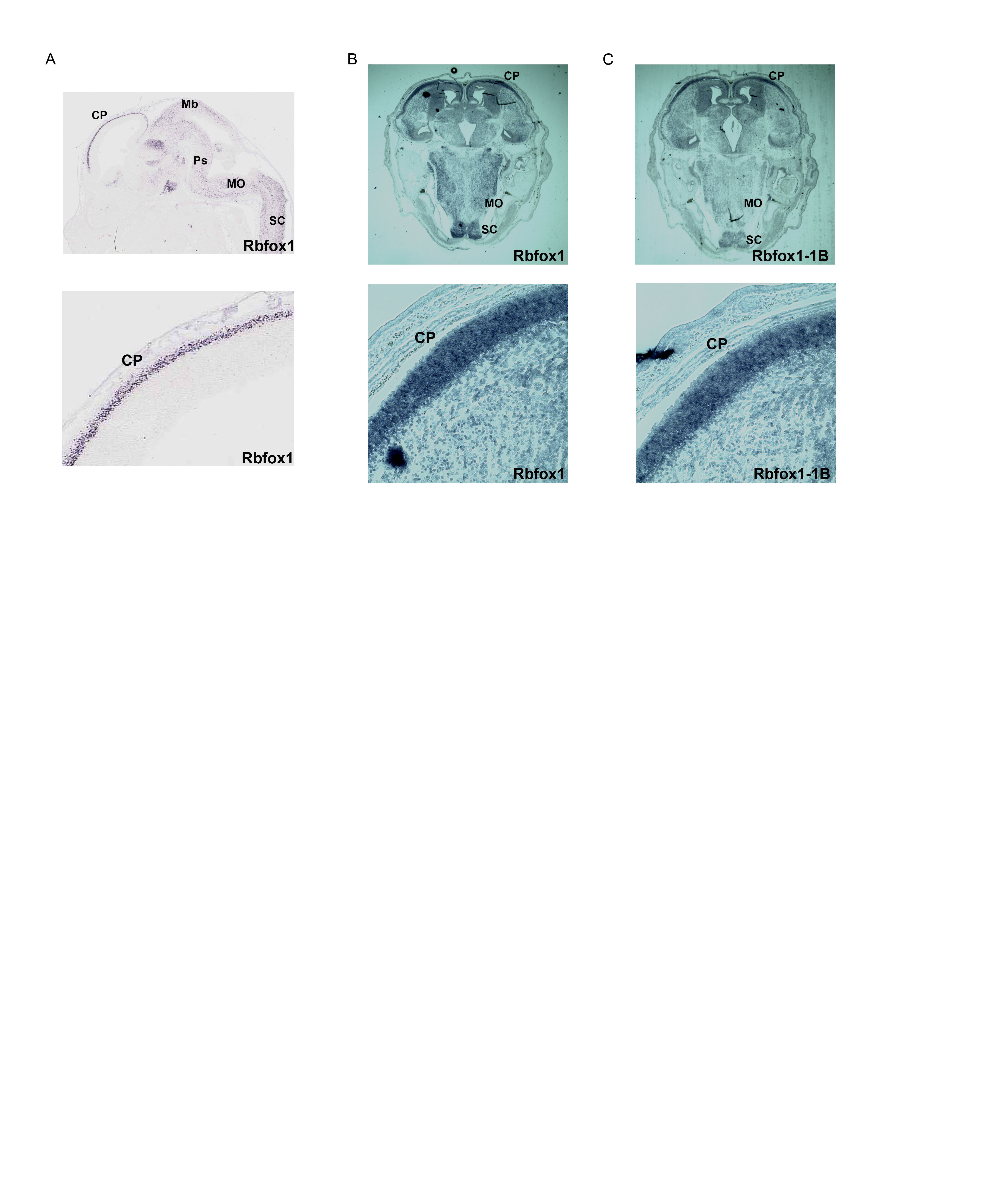

Supplement: FIGURE S3 — Rbfox1 first exon 1B is expressed in the cortical plate of the mouse embryo. (A) In situ hybridization of Rbfox1 (genepaint.org) showing Rbfox1 expression in the cortical plate, and several other brain regions. (B,C) In situ hybridization carried out with probes corresponding to total Rbfox1 (B) or Rbfox1 1B (C). A high expression level of Rbfox1 1B was detected in the cortical plate. [file Image_3.jpg]

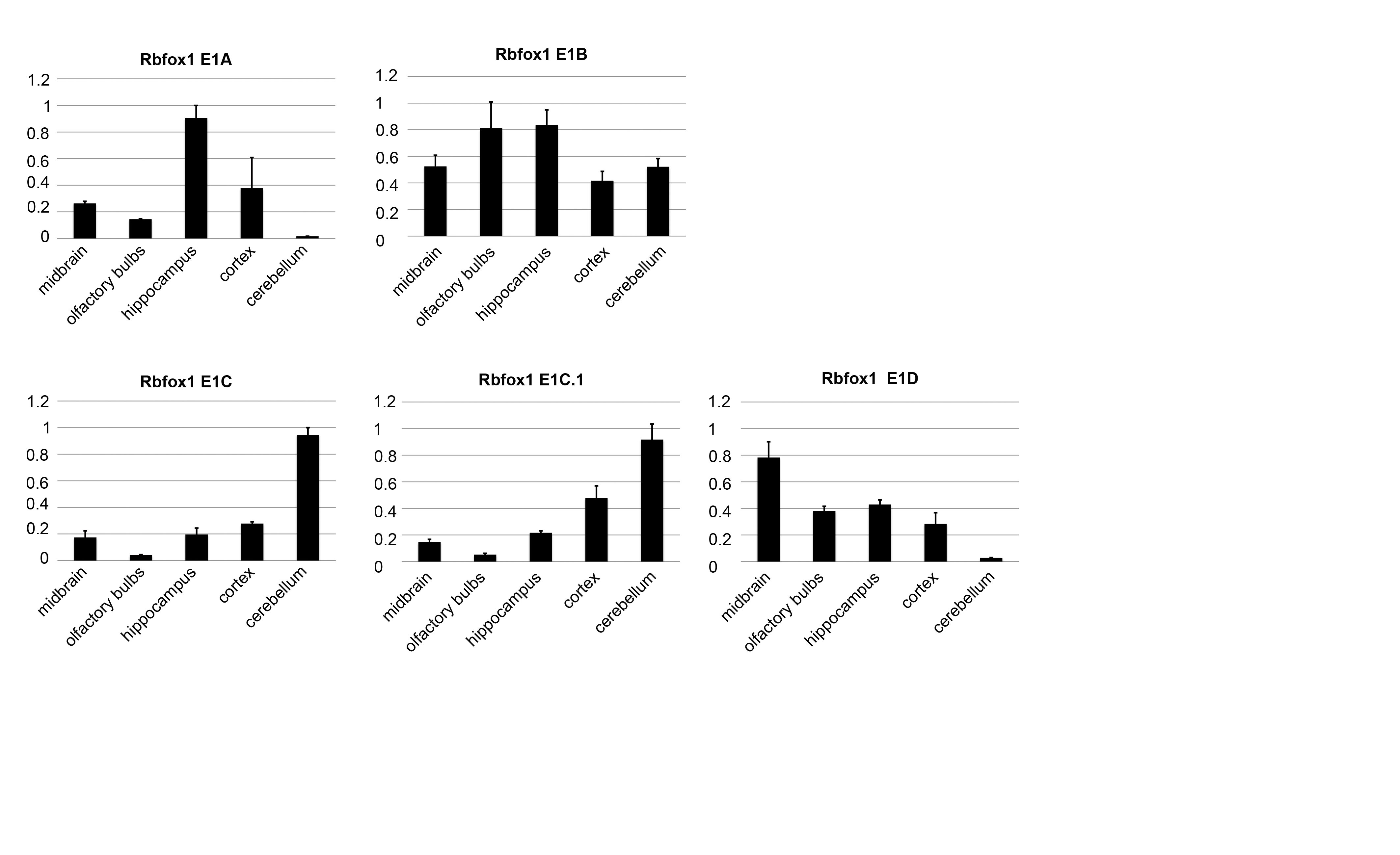

Supplement: FIGURE S4 — Expression of the Rbfox1 alternative first exons in different subregions of the juvenile brain. RT-qPCR with primers corresponding to Rbfox1 exons 20/21 or the alternative first exons. Total RNA was isolated from different brain subregions of 6-weeks old mice, and cDNA synthesis and qPCR were performed; mRNA expression was normalized to Gapdh. [file Image_4.jpg]
