# Supplementary material for: Rbfox1 Is Expressed in the Mouse Brain in the Form of Multiple Transcript Variants and Contains Functional E Boxes in Its Alternative Promoters
Source: Front Mol Neurosci. 2020 May 5;13:66. doi: 10.3389/fnmol.2020.00066 (PMC7214753; doi:10.3389/fnmol.2020.00066)
Supplement: TABLE S1 — Primer sequences. [file Table_1.pdf]

# Table S1: Primer sequences

| Application | Primer | Sequenz (5'-3') | Roche probe |
|---|---|---|---|
| qPCR | Rbfox1_F | GACCCCTACCACCACACACT | 19 |
| | Rbfox1_R | TCTTGGCATCGGTCAAGG | |
| | Rbfox1_alt3end_R | TGCAGCTTAGCTCCTCTGAAGT | |
| | Rbfox1_E1A_F | gtggcgaaggcagaagtg | 16 |
| | Rbfox1_E1A_R | caggactcaccaaaacctgac | |
| | Rbfox1_E1A.1_F | cggtgagaaaagtaaccagtgtag | |
| | Rbfox1_E1A.1_R | caaaacctgactctccaccag | |
| | Rbfox1_E1B_F | GTTGCCTCTGGTGGATGCTG | 68 |
| | Rbfox1_E1B_R | TGAGCTGTTTCCTAGAACTCCTG | |
| | Rbfox1_E1C.1_F | CGGATCAGAATCCGGACCT | 58 |
| | Rbfox1_E1C.1_R | CTTTGATATCCTACCCTGTGTAA | |
| | Rbfox1_E1C_F | ccaatgcgatgtagcaagag | 15 |
| | Rbfox1_E1C_R | gagtcgcctttgatatcctga | |
| | Rbfox1_E1D_F | GCCTTGACTTTTATGTACTTACAGCAG | 11 |
| | Rbfox1_E1D_R | ATAAGGCTGAGCCATTGTGTC | |
| | Gapdh_F | TGCTGAGTATGTCGTGGAGTCT | 157 |
| | Gapdh_R | TTGGCTCCACCCTTCAAGT | |
| | Bcas2_F | CAACCGATTGAATTACTCAGCA | - |
| | Bcas2_R | ACACATTCTTGCCATGCAGT | |
| Cloning | Rbfox1_Prom1B_KpnI_F | ACTGGGTACCAGACGCCTGGCTCTTGATAA | |
| | Rbfox1_Prom1B_HindIII_R | ACTGAAGCTTTCCTCCTGGATTCCTCAGTT | |
| | Rbfox1_Prom1C_XhoI_F | ACTGCTCGAGTACCTCGTGGGGGATATGAA | |
| | Rbfox1_Prom1C_ext_Bglll_R | ACTGAGATCTCATCTTCTTTGCAAAATCCAG | |
| | Rbfox1_Prom1D_XhoI_F | ACTGCTCGAGAGACTGTAGTTTTTTCCAGT | |
| | Rbfox1_Prom1D_BglII_R | ACTGAGATCTCCGATTATCTAACTCACACA | |
| In vitro mutagenesis | Prom1B_ c1658t_a1659t_F | CAGTGCAAACTATGTAAGCAATTCATGGTAGTGTGACCTGAGTA | |
| | Prom1B_c1658t_a1659t_R | TACTCAGGTCACACTACCATGAATTGCTTACATAGTTTGCACTG | |
| | Prom1B_c2865t_a2866t_F | GAGTCGCTCAGCTCGTTTCTGGAGGGGACAGC | |
| | Prom1B_c2865t_a2866t_R | GCTGTCCCCTCCAGAAACGAGCTGAGCGACTC | |
| | Prom1B_c2378t_a2379t_F | CACAGAGAGACACACACACATTCATGCACACATTTTTCTCGC | |
| | Prom1B_c2378t_a2379t_R | GCGAGAAAAATGTGTGCATGAATGTGTGTGTGTCTCTCTGTG | |
| | Prom1B_c2815t_a2816t_c2817t_F | CCAGCCACAGGACCTTTGCGTGCGCCTCGCG | |
| | Prom1B_c2815t_a2816t_c2817t_R | CGCGAGGCGCACGCAAAGGTCCTGTGGCTGG | |
| | Prom1C_c332t_a333t_F | CATCAGTTTGCTCACTTTCTTAGTGCTTGGCAAAGGGGGT | |
| | Prom1C_c332t_a333t_R | ACCCCCTTTGCCAAGCACTAAGAAAGTGAGCAAACTGATG | |
| | Prom1C_c194t_a195t_F | CATTGCTCCTGAGTTAAAATTGGTTGTTTAGTGAGGACTCTGG | |
| | Prom1C_c194t_a195t_R | CCAGAGTCCTCACTAAACAACCAATTTTAACTCAGGAGCAATG | |
| | Prom1C_c236t_a237t_F | GGGACTGTGCAAGGTGCCCTATTCTGTTTTGTGGTACATATTT | |
| | Prom1C_c236t_a237t_R | AAATATGTACCACAAAACAGAATAGGGCACCTTGCACAGTCCC | |
| | Prom1C_c564t_a565t_F | TCGCTTGTTCAAGGCCTTGAATTCCTGCTGGTGGCT | |
| | Prom1C_c564t_a565t_R | AGCCACCAGCAGGAATTCAAGGCCTTGAACAAGCGA | |
| | Prom1C_c652t_a653t_c654t_F | TAGAATCATTGGCATTGCTAGTTTGTGGGTACCATTCTCTGCTGC | |
| | Prom1C_c652t_a653t_c654t_R | GCAGCAGAGAATGGTACCCACAAACTAGCAATGCCAATGATTCTA | |
| | Prom1Cc278t_a279t_c280t_F | AAGAACAAGGGAAGAGATTTGTGCTGGGGGGGGGGTG | |
| | Prom1C_c278t_a279t_c280t_R | CACCCCCCCCCCAGCACAAATCTCTTCCCTTGTTCTT | |
