# Supplementary material for: Rbfox1 Is Expressed in the Mouse Brain in the Form of Multiple Transcript Variants and Contains Functional E Boxes in Its Alternative Promoters
Source: Front Mol Neurosci. 2020 May 5;13:66. doi: 10.3389/fnmol.2020.00066 (PMC7214753; doi:10.3389/fnmol.2020.00066)
Supplement: Supplementary file 6 [file Data_Sheet_1.docx]

**Supplementary Methods**

**In situ hybridization**

Two riboprobes, one corresponding to exon 1B and the other one to a part of the coding sequence of *Rbfox1* were generated by amplifying the respective cDNA fragments by PCR (primers are given in supplemental table 1) and cloning them into the pGEM® T Easy vector (Promega, Mannheim, Germany). After plasmid linearization DIG labeled probes were generated by in vitro transcription using DIG RNA labeling mix and T7 RNA polymerase (Roche, Penzberg, Germany).

Probe hybridization was carried out on E15.5 mouse brain sections (ZYAGEN, San Diego, CA, USA). After dewaxing sections were rehydrated, refixed in 4% paraformaldehyde and proteinase K digested (Roche, Penzberg, Germany) for 10 min followed by washing in PBT (PBS 0,1% Tween 20) for 2 and 5 min. Subsequently, sections were refixed in 4% paraformaldehyde for 5 min, washed in PBT twice for 5 min, acetylated in TAE for 15 min and again washed twice in PBT for 10 min. After air-drying, sections were covered with prehybridization mix and incubated at 65°C for 1 -3 h. Meanwhile, Dig labeled probes (3 µl) were mixed with 300 µl of hybridization buffer and incubated at 85°C for 5 min. The hybridization mix was then added to the sections which were covered with a coverslip and incubated at 65°C over night in a wet chamber. For removal of the coverslips sections were washed with 5x SSC on the next day and then incubated successively in 2x SSC/50% formamide at 65 °C for 30 min, TNE at 37 °C for 10 min, TNE with RNAse at 37 °C for 10 min, TNE at 37 °C for 10 min, 2x SSC at 65 °C for 30 min and twice with 0,2x SSC at 65 °C for 30 min. After cooling down to room temperature sections were washed twice for 5 min with MABT. Subsequently, sections were incubated with 500 µl blocking solution for 1 -2 h and then with 300 µl antibody solution (covered with a coverslip) in a wet chamber over night at 4°C. On the next day sections were rinsed with MABT to remove coverslips and washed twice with MABT for 5 and 10 min. Sections were then washed with NTMT for 10 min and incubated with BM Purple (Roche, Penzberg, Germany) for several days. Finally, sections were washed with NTMT for 10 min and twice with PBS for 5 min each, and then refixed in 4% paraformaldehyde for 30 min. After two washes in PBS each for 5 min the sections were rinsed in water and embedded in Kaisers Glycerol gelatine. In situ hybridizations were analyzed with an EVOS XL Core Cell Imaging System (ThermoFisher Scientific, Rockford, IL, USA).

**Luciferase reporter assays and immunofluorescence experiments**

For luciferase reporter assays 4x10^5^ neurons/well were seeded into 24-well plates and transfected at DIV3 with 4 µg/well of the Rbfox1-luciferase reporter constructs together with 1 µg/well of the renilla control plasmid pRL-TK using Lipofectamine 2000 reagent. Neurons were harvested 24 h later and firefly/renilla luciferase activity was measured on a Centro XS3 LB 960 Microplate Luminometer (Berthold Technologies, Bad Wildbad, Germany.

For immunofluorescent stainings, 8x10^4^ N2A cells and 8x10^5^ primary cortical neurons were seeded into 12- well plates on cover slips and transfected with the Rbfox1 constructs using Lipofectamine2000 reagent (Thermo Fisher Scientific, Rockford, IL, USA).

Twenty-four hours post transfection cells were fixed with 4% PFA for 10 minutes. Subsequently, cells were washed three times 5 minutes with PBS 0.2% Triton and blocked at RT for 30 minutes with PBS 0.2% Triton, 2% sheep serum. The primary antibodies (mouse anti-Rbfox1 (MABE985, Merck/Millipore, Burlington, MA, USA) and rabbit anti-GFP (632592, Clontech, Mountain View, CA, USA)) were incubated in PBS, 2% sheep serum at RT for 1 h in the dark. Before secondary antibody incubation, cells were washed three times 5 minutes with PBS 0.2% Triton. Secondary antibodies (goat anti-rabbit IgG AF488(A27034, Invitrogen, Waltham, MA, USA) and goat anti-mouse IgG AF595(A11032, Invitrogen, Waltham, MA, USA)) were incubated in PBS, 2% sheep serum at RT for 30 minutes in the dark. After antibody incubation, cells were washed three times 5 minutes with PBS 0.2% Triton and mounted with Fluoromount including DAPI (00-4959-52, Thermo Fisher Scientific, Waltham, MA, USA).

**Iso-Seq Sequencing results and analysis**

The sequencing of three SMRT cells was carried out on a Pacific Biosciences (PacBio) Sequel sequencing system. Analysis of the raw reads from three sequencing runs was performed using PacBio SMRT Link v5.1.0 Iso-Seq pipeline using the default parameters (http://www.pacb.com/products-and-services/analytical-software/smrt-analysis/). Brief description about the SMRT Link v5.1.0 Iso-Seq pipeline, first the circular consensus (CCS) reads with a minimum length of 50 bp were generated and classified into full-length (FL) and non-FL (nFL) reads. The classified full-length reads were then clustered at the isoform level into high-quality (HQ) and low-quality (LQ) transcript isoforms.

Two different mapping strategies were used for the downstream analysis. First the high quality full-length reads were mapped against mouse genome (mm10) using GMAP (Wu and Watanabe 2005) and second the obtained high and low quality full-length reads were combined and mapped to the mouse genome (mm10) using STARlong v2.5.3 (Dobin et al. 2013). The parameters used for STARlong alignment is available in the supplementary data. Lastly, the alignments of the transcript were observed and curated using Integrative Genomics Viewer (IGV) v2.3.83 (Robinson et al. 2011).

| **Sequencing results** |  |  |  |  |
| --- | --- | --- | --- | --- |
| **SMRT Cell ID** | **Reads** | **Yield** | **Polymerase Read N50** | **Subread N50** |
| 1 | 751,773 | 8.49 Gbp | 28 kb | 3.2 kb |
| 2 | 639,157 | 4.83 Gbp | 18 kb | 2.8 kb |
| 3 | 742,692 | 7.77 Gbp | 28 kb | 3.3 kb |
|  |  |  |  |  |
| **Isoform analysis results** |  |  |  |  |
| HQ isoforms | 44,988 |  |  |  |
| HQ isoforms mean length | 2,259 bp |  |  |  |
| Mapping rate using GMAP | 99% |  |  |  |
| Mapping rate using STARlong | 63% |  |  |  |

**STARlong parameters**

--runMode alignReads

--outSAMattributes NH HI NM MD

--readNameSeparator space

--outFilterMultimapScoreRange 1

--outFilterMismatchNmax 2000

--scoreGapNoncan -20

--scoreGapGCAG -4

--scoreGapATAC -8

--scoreDelOpen -1

--scoreDelBase -1

--scoreInsOpen -1

--scoreInsBase -1

--alignEndsType Local

--seedSearchStartLmax 50

--seedPerReadNmax 100000

--seedPerWindowNmax 1000

--alignTranscriptsPerReadNmax 100000

--alignTranscriptsPerWindowNmax 10000

**References**

Dobin, A., Davis, C.A., Schlesinger, F., Drenkow, J., Zaleski, C., Jha, S., Batut, P., Chaisson, M., and Gingeras, T.R. (2013). STAR: ultrafast universal RNA-seq aligner. *Bioinformatics* 29**,** 15-21.

Wu, T.D., and Watanabe, C.K. (2005). GMAP: a genomic mapping and alignment program for mRNA and EST sequences. *Bioinformatics* 21**,** 1859-1875.
